# Gender-Biased Expectations of Altruism in Adolescents

**DOI:** 10.3389/fpsyg.2018.00484

**Published:** 2018-04-10

**Authors:** Mauricio Salgado

**Affiliations:** Centre for Research in Inclusive Education, School of Sociology, Universidad Andres Bello, Santiago, Chile

**Keywords:** adolescents, altruism, dictator games, gender beliefs, gender roles

## Abstract

Research suggests that women, but not men, manifest gender-biased expectations of altruism: while women expect other women to be more altruistic, men expect women to be as generous as men. Do adolescents expect women and men to behave differently regarding altruism? I analyse adolescents' gender beliefs about altruism using a modified Dictator Game. Results indicate that adolescents believe that others of same gender are more altruistic than others of the opposite gender. I also found that adolescents' agreement with the existence of different societal roles for men and women moderates the relationship between gender and gender beliefs. Although it was expected that adolescents who agree with different gender roles would expect women to be more generous, surprisingly, the results presented here confirm this only for male adolescents, but in the opposite direction: the more male adolescents agree with the existence of different gender roles, the more they seem to believe that men are more generous than women. Meanwhile, female adolescents believe that women are more altruistic unconditionally. Thus, the previously documented bias seems to be already in place during adolescence, above and beyond other confounding factors. Adolescents' in-group bias, and their socialization into different cultural values regarding gender roles are discussed as potential explanatory mechanisms for these gender beliefs.

## Introduction

There is increasing interest in investigating the extent to which men and women differ in behavior and beliefs, and a great bulk of research has explored and documented these differences. Simple economic games, such as the dictator game (hereafter DG) have provided an avenue for examining the impact of gender on *altruistic behavior*–when individuals allocate material or symbolic payoffs to others at a personal cost (Kahneman et al., 1986; Forsythe et al., 1994). The general setting of DG allows researchers to study not only altruistic disparities between men and women (i.e., differences in revealed social preferences), but also to explore differences in *gender beliefs*. Gender beliefs are defined as subjects' expectations concerning how women and men behave, and how women and men should behave (Heilman, 2001). Gender beliefs are individuals' stereotypes and also cultural artifacts: rules or instructions for enacting gender that result in an asymmetrical distribution of resources between men and women in society (Ridgeway and Correll, 2004). These beliefs act as a background frame that biases our behavior, and our expectations of others' behavior, in gender-consistent directions. The salience of these gender beliefs seems to be of paramount importance regarding altruistic (or selfish) behaviors in men and women. For instance, previous research in adults, using the general framework of the DG, has shown that women, but not men, expect other women to be more altruistic (Aguiar et al., 2009; Brañas-Garza et al., 2016). That is, only women manifest gender-biased expectations of altruism.

In this paper, I use a modified version of the DG to study adolescents' gender beliefs of altruism. I wanted to examine two clear and straightforward questions: First, do adolescents hold special beliefs for females regarding altruism? Second, are these gender beliefs moderated by their agreement with different gender roles in society? To answer these questions, a sample of Chilean high-school pupils participated in a two-stage study. In the first stage, adolescents took part in a DG. In the second stage, they played a modified DG for studying gender beliefs of altruism. Only the second stage of the study is analyzed in this paper. Two main hypotheses directed this study. First, as previous research has shown for adults, here it is also predicted that a relationship exists between adolescents' gender and their gender beliefs of altruism. Second, it is also predicted that adolescents' degree of endorsement of different gender roles for men and women in society provides the boundary conditions for the relationship between adolescents' gender and their gender beliefs of altruism. The rest of the paper is structured as follows: Dictator games and gender beliefs presents a literature review on DGs and gender beliefs. The context in which this study was carried out (Chile) is presented in the Chilean context, while materials and methods explains the experimental design implemented in this research. Data analysis and main results are described in result. A discussion of the experimental results is presented in discussion.

## Dictator games and gender beliefs

The DG gets its name from the fact that player *i* (the dictator) unilaterally determines the distribution of a monetary or symbolic pie between herself and player –*i* (the recipient). While the dictator may divide the pie in the manner she sees fit, the recipient may not make any claim to the pie and has no sanctioning power over the dictator's distribution. Theoretically, the solution is obvious: player *i* keeps all and player –*i* receives nothing. However, results from different cultural contexts depart from this predicted selfish behavior: more than 60% of dictators allocate a positive amount to recipients, giving away 25% on average of the given pie (Henrich et al., 2004; Levitt and List, 2007). This sharing of the pie is interpreted as altruism, since the dictator provides the recipient a benefit at a personal cost (i.e., the dictator's net gain suffers).

Evidence on whether children and adolescents are altruistic, and whether they exhibit gender differences of allocations in DG has yielded inconsistent results. Harbaugh and Krause (2000) found that children's allocations in DGs (between 6 and 12 years old) are in line with those documented in adults, and that they give away an average of 29% of their initial endowment. Regarding gender differences, Harbaugh et al. (2003) carried out a study in which subjects aged 7–18 years old played the DG. They found that boys tended to be less altruistic than girls, and this difference increased with age. However, Benenson et al. (2007), with younger children, aged 4, 6, and 9 years, found no significant differences between genders. Instead, they found a significant effect of socioeconomic status (SES) on children's altruism. In their study, children from higher SES environments behaved more altruistically. More recently, Dreber et al. (2013) found that in their sample of adolescents aged 16–18 years old, girls were more altruistic than boys in the DG.

Literature on whether young children's altruism results from the influence of social environments or whether it is innate seems to be contentious (Warneken et al., 2007; Fehr et al., 2008; Olson and Spelke, 2008). However, young children behave more selfishly than adolescents in DGs, so allocations by children become more egalitarian with age. Henrich et al. (2005) research in 15 small societies concluded that altruism is learned slowly over the first two decades of life, and subsequently changes little. This fact stresses the fundamental role in shaping individual altruism of early *socialization*: the internalization of local cultural norms and values that are incorporated into individual preference functions during ontogeny (Gintis and Helbing, 2015).

Therefore, altruistic behavior in adolescents seems to be dependent on the normative judgements they acquire through socialization. It has also been documented a relationship between adolescents' altruism and the internalization of hegemonic gender-roles. Based on longitudinal data, Fan and Marini (2000) documented that the gender-role attitudes of young women and men are linked to their value orientations, which in turn derive from socialization from family and is influenced by gender. Previous studies also suggest that gender-specific socialization practices lead to gender differences in altruistic modes of reasoning and behavior (Carlo et al., 1996). Thus, there is no reason to assume that adolescent gender beliefs are independent of their judgements regarding gender roles in society.

Gender disparities in altruistic behavior have been explained within the *Social Heuristics Hypothesis* (SHH) (Rand et al., 2016). The SHH says that social strategies which are successful in daily life become automatized as intuitive responses. Although altruism is not advantageous, if being selfish is seen negatively by others, it may create reputational costs. If so, then altruism could payoff in the long run. According to SHH, since successful strategies are intuitive, intuition should favor altruism for most people. However, it may not be the case that all people are harmed from being selfish, such that moderators may exist for whether altruism is advantageous and thus favored by intuition. Gender seems to be a compelling moderator of altruism. Stereotypes concerning gender roles –such that women are expected to be communal, “homemakers” (i.e., unselfish and concerned with others) while men are expected to be agentic, “breadwinners” (i.e., self-assertive and independent)– and the fact that women disproportionately occupy roles that mandate self-sacrificing and altruistic behavior –such that women habituate to be altruistic– might reinforce altruistic behaviors in women more than men (Rand, 2017). Because women occupy more roles that demand communal behaviors, such tendencies become stereotypic of women and are incorporated into a female gender role.

The SHH is compatible with two traditional approaches to gender stereotypes. In economic literature, *Statistical Discrimination Theory* (SDT) (Arrow, 1973) claims that, in the absence of direct information, group membership serves as cheap information on characteristics that are difficult to observe, so a decision maker would substitute group averages (or variances) using visual signals, facilitating behavior. Due to the sexual division of labor, gender might be considered a proxy for altruistic behavior: women could be considered, on average, more generous than men. In the social psychology literature, *Social Role Theory* (SRT) (Eagly et al., 2000) argues the gender beliefs individuals hold about sexes derive from observations of the different gender roles men and women play in society, although these roles reflect the sexual division of labor and the gender hierarchy of the society (Ridgeway and Correll, 2004; Ridgeway and Kricheli-Katz, 2013). Researchers in SRT have long documented that occupational roles are more important than sex in determining beliefs about female communal and male agentic qualities (Eagly and Steffen, 1984). Therefore, gender beliefs individuals hold in a society also predominantly depend on the extent to which women are included and participate in the economy. This socio-cultural feature seems to be particularly important in the Chilean society.

## The chilean context

**Figure 1:**
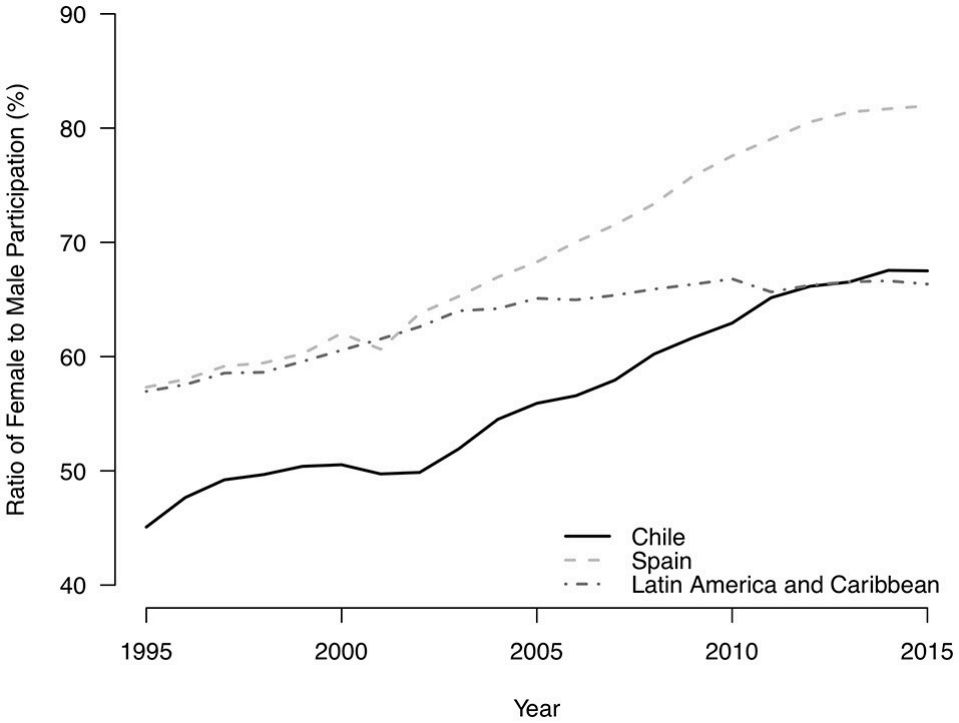
Ratio of female to male labor force participation rate (%). Source: Own elaboration based on The World Bank (2015) statistics.

An important characteristic of the Chilean labor market has been the historically low participation of women in the workforce. As can be seen in Figure 1, Chile's female labor participation rate differs importantly from the those observed in more developed but culturally similar countries, such as Spain, and even from the regional average of Latin America and Caribbean countries (The World Bank, 2015). Although this situation in Chile has been improving during the last two decades, relatively low rates of female participation in the labor market still persist. This situation is even more unusual considering the higher degree of development and high levels of education for women compared with other countries of the region. In addressing this lag, some researchers have found a strong relationship between female labor participation and strong, *machista* cultural traits present in the country. For instance, the Chilean labor market penalizes professional certificates associated with women. Schurch's analysis on the main factors that impact returns of higher education degrees found a “wage punishment” for graduates of female dominated disciplines (Schurch Santana, 2013). Also, Contreras and Plaza (2010) concluded that Chilean women who have more traditional attitudes toward gender roles exhibit lower labor participation, and that this negative association between cultural factors and female participation in the labor market is greater than the effect of the accumulation of human capital. More recently, Mora and Blanco documented several and subtle mechanisms that devalue female participation in the Chilean labor market, such as their “reproductive burden” (Mora and Blanco, 2017).

National surveys show a great mixture of traditional and liberal opinions among Chilean adolescents regarding gender roles and family values. Whereas a majority of adolescents (between 15 and 19 years old) agree that men and women have the same skills to occupy important positions in the government (83%), and that men and women should share equal responsibilities at home (79%), 24% of Chilean adolescents believe that, in general, women with children are more fulfilled than those who do not have children. Also, 54% of adolescents believe that marriage should be for life, and 59% reject legalization of abortion to those who want it (INJUV, 2015). Thus, despite the country's economic growth and sociocultural change during the last decades, Chilean adolescents still exhibit some traditional values regarding family and women rights that might determine their gender expectations of altruistic behavior.

Hence, it can be claimed that gender beliefs emerge from the productive work of sexes; the characteristics embodied in the different roles women and men play in the economy become stereotypic of women or men. These gender beliefs also comprise distinctive ways in which women and men are expected to behave, particularly regarding altruism. Because of the relatively low female participation in the economy, and the traditional values regarding women and family still present in the Chilean society, in this paper I conjecture that Chilean adolescents who endorse the norm that men and women have different roles to play in society might also expect that men and women behave in stereotypic ways. Along the lines of the gender roles distinction between men as “breadwinner” and women as “homemaker,” adolescents who strongly agree with gender roles should expect women to be more altruistic than men. Therefore, I predict that adolescents' degree of endorsement of different gender roles in society provides the boundary conditions for the relationship between adolescents' gender and their gender beliefs about altruism.

## Materials and methods

The present study was carried out on a web-based system. Adolescents had to accept an informed assent form, and they had to bring a signed parental consent to participate in this study. I carried out 49 experimental sessions in 22 schools, from which participants were recruited in three different regions of Chile: Valparaíso, Araucanía, and Metropolitana regions. Each session with a group of 8 or 10 pupils was conducted at the school's computer laboratories. One member of the research team led the sessions; briefing the adolescents about the study, reading the activity instructions^1^ (also displayed on screen), and answering participant questions. The experimenter explained that their participation was voluntary, and their answers would remain anonymous (a double-blind procedure was followed). Participants were not allowed to speak to one another. The sessions lasted for approximately 45 min.

Due to ethical concerns from the author's institutional *Research Ethics Committee*, participants did not receive real money in return for joining in the activities. Pupils were given tokens instead, symbolized on screen as golden coins. Although the evidence indicates that in DGs subjects give more when handling real money (Engel, 2011), research with children and adolescents has shown that tokens are an appropriate resource and perform well (Harbaugh and Krause, 2000; Benenson et al., 2007). In the present study, participants amassed their earned tokens in a virtual, personal “bank account.” The bank balance was always displayed on screen during the activities, and it was updated automatically according to the participants' performance during the activities. At the end of the session, the computer system informed participants their total amount of earned tokens, which they could trade for bags of popular snacks^2^ priced $2,500 Chilean pesos (US $3.5 dollars approximately), $2,000 (around US$2.7 dollars), and $1,000 (around US$1.5 dollars). These rewards were shown to participants before the sessions began. It was clear that these payoffs were sufficient to engage the interest of participants.

Experiments were conducted in two stages. In the first stage, participants played a four-round DG, with an endowment of 10 electronic tokens for each round. This four-round DG provided participants the experience needed to understand the next stage of the experiment. In the second stage of the experiment, all subjects participated in a guessing activity which aimed at registering adolescents' gender beliefs of altruistic behavior. Only the results of this second stage are analyzed here.

Participants also completed a socio-demographic survey and a gambling task to measure participants' risk preferences before the experiments. The survey was used to obtain participants' sociodemographic information (e.g., gender, age, ethnicity), home possessions (10 items), highest educational level reached by the head of the household (8 categories). I applied standard scores to the 10 home resources and to the educational level reached by the head of household, so I could compute a (standardized) scale of adolescents' socioeconomic status (z-SES, alpha = 0.78). A list of all socio-demographic variables with question wording and descriptive statistics can be found in Table A1 in the Appendix. Also, there was question measuring the degree to which adolescents in the sample endorsed one very specific normative declaration. This item, named *Gender Roles*, gauged participant degree of agreement with the statement “*Men and women each have different roles to play in society*.” Adolescents rated these moral judgement statements on a 6-point Likert scale, from *strongly disagree* (= 0) to *strongly agree* (= 5).

It was important to control for risk-sensitivity in this study, since the experimental design involved the drawing of lots for different prices. Thus, it could not be assumed that all participants are risk-neutral in choosing the men or women box. Therefore, I adapted a gambling task designed by Ashraf et al. (2006) to measure adolescents' risk-seeking preferences. In this activity, adolescents were confronted with a choice between a safe option (to earn a certain number of tokens) and a gamble option that consisted of two possible outcomes (to earn 15 tokens or 0 tokens) with equal probability. Participants played the gambling task through six rounds, and each round increased the outcome of the safe option by 1 token, from 5 to 10, whereas the two possible outcomes of the gamble option were constant. Thus, participants experienced a dynamic environment that changed their level of risk exposure: while in the first three rounds the expected value of the gamble option is higher than the safe option, the opposite is true for the three last rounds. To model the *i*th participant's risk-seeking score α, I used the following score function:

$$\begin{array}{l} \alpha_{i}=\overset{6}{\underset{j=1}{\sum }}e^{\frac{R_{i}\times j}{\lambda }}-1 \end{array}$$

where *R* equals 1 if participant *i* chooses the gamble option (and 0 otherwise), *j* is the number of the round in which the decision is made, and λ is a constant parameter to keep α scores between the values 0 (= maximum risk aversion) and 6 (= maximum risk seeking).

After completing the questionnaires and the gambling task, adolescents took part in a guessing activity to earn tokens. I adapted a previous design to study gender beliefs of altruism (Aguiar et al., 2009), which follows the general framework of the DG. In this activity, two different boxes labeled “women” and “men” were displayed on screen. Participants were told that each box contained 20 allocations made by each of 40 dictators (20 women + 20 men), obtained in a previous DG in another school with pupils of similar age and characteristics. The only decision participants had to make was to select on-screen the box they preferred (by clicking on them), either the “women” or the “men” box. Then, the computer system would randomly choose one dictator's allocation from the selected box and would display it on screen. The displayed allocation corresponded to the number of tokens each participant earned, so it was immediately added to their virtual bank account. I assumed that participants wanted to maximize their payoffs and, therefore, would choose the box in which they expect to obtain a higher average payoff. Hence, adolescents' choices would reveal their beliefs about which gender they expected to be more altruistic in DGs. In the present study, I followed research design (Aguiar et al., 2009), but I added additional control variables and a different analytical strategy.

In total, 488 pupils participated in this study. Data from 3 participants were excluded because they declared to be more than 20 years old. The final sample included 485 high-school pupils, 42% women, between the ages of 15 and 20 (*M* = 16.93, *SD* = 1.17). 37% of the pupils attended single-sex schools: 119 boys-only schools, 58 girls-only schools. 14% of the sample declared to belong to one of the Chilean indigenous groups. Fifty-one percent of the participants' heads of households had completed higher education. Regarding socioeconomic status, 5.8% of the young participants were low SES; 14.2% medium-low SES; 34.6% medium SES; 28.9% medium-high SES; and 16.5% were high SES. On average, boys scored slightly higher (*M* = 3.6, *SD* = 1.78) than girls (*M* = 3.42, *SD* = 1.77) in risk seeking, but this difference was not significant (assuming equal variances, *p* = 0.67), *t*_(483)_ = 0.96, *p* = 0.34.

## Results

Table 1 summarizes the main results of the experiment, showing the number of female participants that chose either women or men and the number of male participants subjected to the same choice. The Pearson's χ^2^ test supports the hypothesis that adolescent gender is associated with the choice of the box, χ^2^(1) = 57.33, *p* < 0.001. However, the frequency distribution indicates an important difference compared to previous research. It is observed in Table 1 that adolescents in this experiment tended to choose dictators of *similar* gender to them, although this effect was slightly stronger for female adolescents. Thus, most female adolescents picked the women box, while most male participants chose the men box. This result differs from that obtained in Aguiar et al. (2009), which detected that women believed that women were more altruistic, but men believed that women were as altruistic as men. In the experiment reported here, the results indicate the presence of what can be identified as *gender-biased expectations* of altruistic behavior among adolescents in Chile. Thus, these results confirm the predicted relationship between adolescents' gender and their gender beliefs of altruism.

**Table 1:** Participant decisions in the experiment. Relative frequencies in rows.

		**Gender chosen**		**Total**
		**Women**	**Men**	
Participants' Gender	Female	142 (70.0%)	61	203
	Male	99	183 (64.9%)	282
	Total	241 (49.7%)	244	485

Based on the results in Table 1, I estimated two focused comparisons: Firstly, the odds of revealing gender-biased expectations of altruism in the election of the box; and secondly, the odds of choosing the women box. First, the odds of participants choosing dictators of the same gender to them were 26% higher (CI 95% [0.86, 1.85]) if the participants were females than if they were males. Thus, gender-biased expectations of altruism were somehow stronger among female adolescents. Results also show that the odds of participants choosing female dictators were 4.3 times higher (95% CI [2.92, 6.34]) if the participants were females than if they were males. Both focused comparisons provide strong evidence that female and male adolescents hold strong gender-biased expectations of altruism. To understand the relationship between the adolescents' characteristics and the likelihood of choosing the women box in the experiment (i.e., choosing the female dictators), I estimated several logistic regression models to gauge the direct and moderation effects of the predictors on the response variable^3^. The results from these analyses are shown in Table 2.

**Table 2:** Coefficients of the logistic models predicting whether an adolescent choose the women box (i.e., adolescents prefer female dictators).

	**Model 0**		**Model 1**		**Model 2**		**Model 3**		**Model 4**	
**Included**	***b***	***S.E***.	***b***	***S.E***.	***b***	***S.E***.	***b***	***S.E***.	***b***	***S.E***.
Adolescents' gender (Male = 0)	1.46^***^	0.20	1.56^***^	0.21	1.53^***^	0.21	1.05^***^	0.29	1.05^***^	0.29
Adolescents' SES (Z-scores)			0.23	0.19	0.19	0.19	0.22	0.20	0.22	0.20
Risk-seeking score			0.01	0.06	0.01	0.06	0.01	0.06	0.01	0.06
Ethnicity (Not Amerindian = 0)			−0.12	0.30	−0.12	0.30	−0.13	0.30	−0.14	0.30
Age (centered at grand mean = 16.93 years)			−0.15^#^	0.09	−0.15^#^	0.09	−0.14	0.09	−0.14	0.09
Gender roles					−0.08	0.05	−0.20^**^	0.08	−0.20^**^	0.08
Gender roles ^*^ Adolescents' gender							0.27^*^	0.11	0.27^*^	0.11
Single-gender school? (No = 0)									0.03	0.20
Constant	−0.61^***^	0.12	−0.69^**^	0.24	−0.51	0.27^#^	−0.30	0.28	−0.31	0.30
Number of cases	485		485		485		485		485	
Log likelihood	-306.86		-303.64		-302.60		-299.68		-299.67	
Pseudo *R*^2^	0.09		0.10		0.10		0.11		0.11	

# *p < 0.1*;

* *p < 0.05*,

** p < 0.01;

*** *p < 0.001*.

Table 2 starts with a baseline model (Model 0) that includes only one predictor: adolescents' gender. This baseline model reproduces the results presented in Table 1, and it shows that gender is significantly correlated with the choice of the female box: female adolescents are more likely to choose the women box than male adolescents. Thus, Model 0 predicts that 35% of male adolescents would prefer a female dictator, but 70% of women would favor a female dictator. The rest of the models in Table 2 estimate the relationship between adolescents' gender and the outcome variable, when controlling for potentially confounding variables.

Model 1 adds the participants' risk preferences score (the higher the score, the higher the level of risk taking preference) and some demographic variables (i.e., ethnicity, age, SES). Some of these variables have been found to be correlated with adolescent gender beliefs in economic decisions (Pedersen, 1996; Rowley et al., 2007). In Model 1, gender remained positive and strongly correlated with choosing the female box net of all other variables. The relatively large coefficient for gender in Model 1, shows that gender-biased expectations of altruism held by female and male adolescents were very strong, so adolescents' gender significantly correlates with the odds of choosing the box that corresponds to their own gender above and beyond confounding factors. From the rest of the variables, only age had a negative and almost statistically significant relationship. This suggests that the older the adolescents become, the less likely they are expected to choose the women box. In synthesis, after controlling for the other variables, Model 1 predicted that 71% of female adolescents would prefer the women box, but just 34% of males would choose that box. However, a Person's chi-square test of the null hypothesis that the newly included variables in Model 1 compared to the baseline model added nothing to our understanding of the likelihood of choosing the women box has a test statistic value of χ^2^(4) = 6.44, *p* = 0.17, which is insufficient to reject the null hypothesis.

Model 2 added an additional covariate, named Gender Roles, which gauged participants' degree of agreement with the statement “Men and women each have different roles to play in society.” Results from Model 2 show that when controlling for the other variables in the model, the relationship between adolescents' agreement with different gender roles in society and the response variable was negative but not significant. Thus, the variable Gender Roles does not improve our understanding of an adolescent's likelihood of choosing the women box, compared to what was known from Model 1, χ^2^(1) = 2.07, *p* = 0.15.

However, the previous result does not mean that adolescents' normative judgements on different roles for men and women in society are not associated with the manifestation of gender-biased expectations of altruism. This judgement may condition the strength or direction of the relationship between adolescents' gender and the likelihood of expressing gender-biased expectations of altruism. Our research hypothesis predicted that the relationship between adolescents' gender and their gender beliefs of altruism is moderated by their degree of endorsement of different gender roles in society. I tested for this hypothesis in Model 3 by estimating the moderating effect that adolescents' degree of agreement with different roles for women and men in society has on their gender beliefs of altruism. Model 3 suggests that adding this interaction effect significantly increases our understanding of the adolescent's likelihood of choosing the women box, compared to what was known from Model 2, χ^2^(1) = 5.85, *p* = 0.016.

**Figure 2:**
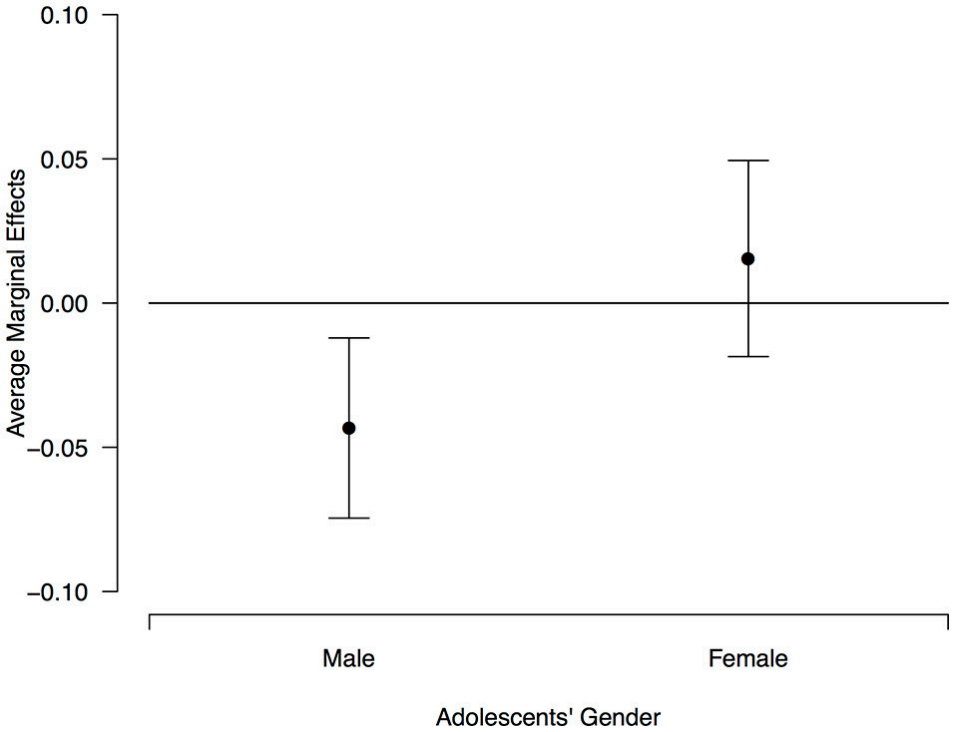
Average marginal effect of degree of agreement with gender roles for male and female adolescents on the predicted probability of choosing the women box. Brackets display 95% confidence intervals.

The coefficients in Model 3 indicate that the effect of adolescents' gender on the likelihood of choosing the women box is moderated by their endorsement of the normative belief that there are different roles to be played by women and men in society. However, the effect of this normative judgement is different for female and male adolescents. Figure 2 displays the (predicted) average marginal effects of gender roles on boys and girls in our experiment. For male adolescents, the average effect of gender roles is negative and significant (the confidence intervals do not cross the zero effect) that is, the more in agreement a male adolescent is with different gender roles in society, the less likely he will choose the women box. For female adolescents, the average effect is statistically non-significant. Therefore, the results of Model 3 indicate that only male adolescents are “sensitive” to the moderating effect of endorsing different gender roles in society in the stated relationship between their gender and their gender beliefs.

**Figure 3:**
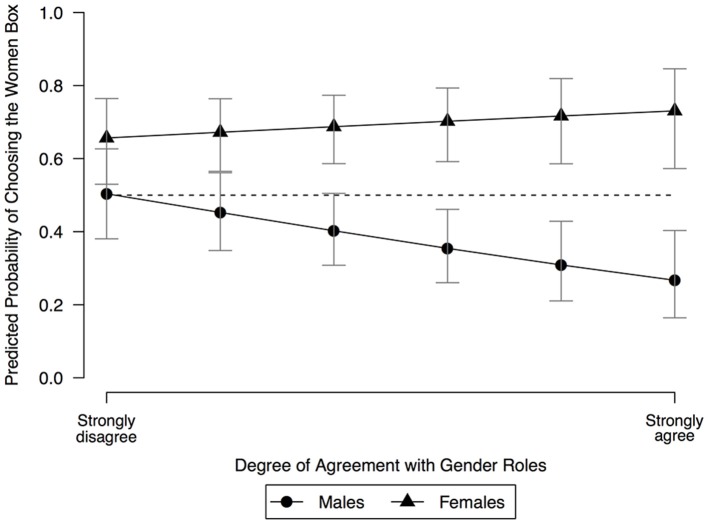
Relationship among three variables: degree of agreement with gender roles, adolescents' gender, and predicted probability of choosing the women box. The dashed line represents the 50% probability. Brackets display 95% confidence intervals.

Figure 3 depicts this moderation effect by computing the predicted probabilities of choosing the women box across the values of the 6-point scale of the adolescents' degree of agreement with gender roles. This was done separately for boys and girls. Figure 3 suggests that the *form* of the relationship between adolescents' gender and their gender beliefs of altruism is conditional upon their degree of agreement with the existence of different roles played by women and men in society (i.e., the curves are non-parallel). Four conclusions were obtained from this analysis. First, the predicted probabilities suggest that the more adolescents agree with different gender roles in society, the stronger the gender-biased expectations of altruism among female and male adolescents will be. Secondly, Figure 3 also suggests that among male adolescents, the moderating effect of this normative judgement is stronger, since the male slope is steeper than the female slope. Thirdly, there seems to be little difference in the likelihood of choosing the women box among female and male adolescents who strongly disagree with different gender roles in society, so these adolescents show less gender-biased expectations of altruism. Finally, female adolescents predicted probabilities of choosing the women box were above the 50% across the values of agreement with gender roles. This is not the case for male adolescents who disagree with the existence of different gender roles in society, whose predicted probabilities of choosing the women box are almost 50%. This last result reinforces the conclusion that female adolescents describe stronger gender-biased expectations, and that these expectations of altruism are unconditional.

Finally, Model 4 (see Table 2) tests the relationship between group-gender composition with the dependent variable, because sitting in a room in which all other decision-makers were of the same (or different) gender could have had important effects through *gender priming*^4^. The log-odds coefficients for this covariate indicates that group-gender composition had no significant relation on the likelihood of choosing the women box. I also tested for and did not find significant interaction effects between adolescents' gender and single-sex schools (not shown here). A chi-square test of the null hypothesis that the included variable in Model 4 –gender-group composition (i.e., *Single-Gender School?*)–, compared to Model 3, added nothing to our understanding of the probability of choosing the women box, having a test statistic value of χ^2^(1) = 0.02, *p* = 0.90, which was insufficient to reject the null hypothesis.

## Discussion

I studied adolescent gender beliefs of altruism in DGs among a group of Chilean adolescents. To do so, I adapted a research design proposed by Aguiar et al. (2009). Results indicate that adolescents manifested strong gender-biased expectations of altruism. This means that both female and male adolescents believed that others of same gender were more altruistic than others of opposite gender. However, this preference was moderated by adolescents' level of endorsement of gender roles in society. Thus, adolescents who highly endorsed the normative expectation that men and women have different roles to play in society manifested stronger gender-biased expectations of altruism. On the contrary, male adolescents who held less naturalized judgements pertaining to gender roles manifested decreased gender-biased expectations of altruism. This moderating effect was not observed in female adolescents, who preferred women dictators regardless of their level of agreement with gender roles. Previous research using the same experimental design with adults (i.e., Spanish undergraduates) indicated that women manifested gender-biased expectations of altruism, but men did not. Surprisingly, the direction of the moderating effect was contrary to what was expected, given the gender-role distinction between women “homemaker” and men “breadwinner” still present in Chilean society. Two potential and complementary explanations are evaluated below.

The gender-biased expectations of altruistic behavior detected in this study make sense in light of *social identity theory* (Tajfel and Turner, 1979; Scheepers et al., 2006). According to this theory, the tendency to favor the in-group over the out-group plays an *identity-expressive* role, which aims at creating, expressing, and thereby confirming a sense of group and personal identity. Since gender is one of the most meaningful social categories in adolescent life (Renk and Creasey, 2003), adolescents might manifest gender-biased expectations of altruism because of this identity-expressive function of in-group bias. The latter means that adolescents reproduce their own identity by expecting that other adolescents like them (i.e., same gender) will behave more altruistically than those of the opposite gender. However, the results presented here also show that this identity-expressive function of in-group bias is not constant, but conditional upon the beliefs regarding gender roles held by female and male adolescents. Adolescents who hold less naturalized beliefs regarding gender roles tend to be indifferent regarding the dictators' gender, although this moderating effect of gender roles appears clearly for male adolescents. Therefore, both cognitive and sociological factors might explain the registered differences between male and female adolescents.

Each social theory, SHH, SDT, and SRT argue that the beliefs people hold about the sexes are derived from the gender division of labor and gender hierarchy of the society. Therefore, gender participation in the labor market might underpin gender stereotypes into a distinction between men “breadwinners” and women “homemakers” (Eagly et al., 2000). Because in Chile female participation in the labor market is relatively low, and it is penalized in terms of wages, there might be stronger gender beliefs regarding altruistic behavior. Besides, Spain is a more developed country than Chile, and development produces not only economic prosperity, but also changes that shape human attitudes and actions (Lipset, 1959; Acemoglu and Robinson, 2001). More specifically, development produces a cultural shift in two dimensions: (1) from traditional to secular-rational values; and (2) from insecure survival to existential security (Inglehart and Welzel, 2010). Indeed, according to the *World Values Survey*, Spain ranks much higher than Chile in those two dimensions (Inglehart and Welzel, 2010). Economic development and higher female participation in the labor market make most adolescents of advanced countries grow in cultures that emphasize values such as autonomy, subjective well-being, and gender equality. Cultures that espouse an egalitarian attitude regarding gender roles are more likely to foster this attitude in their children and youth through socialization. Previous research has found that adolescents' standpoints toward family and gender roles from wealthier countries are less traditional than their peers from developing countries (Gibbons et al., 1991). It is likely then that the cultural values present in Spain lead most individuals to disagree with different gender roles in society; in Chile the opposite trend might be present.

Indeed, when controlling for the moderating effects of adolescents' agreement with different gender roles in society, the results presented here and that carried out in Spain by Aguiar et al. (2009) are similar: among adolescents' who strongly disagree with this judgement, female adolescents expect women to be more altruistic, while male adolescents expect women to be as generous as men. Consequently, the results presented in this paper suggest that the gender-biased expectations of altruistic behavior are not only stronger amongst women, but for that group this bias is less conditional upon socialization to cultural values such as gender equality (present in Spain but not in Chile). Nevertheless, the question remains: why wouldn't more egalitarian gender-roles values affect men and women's gender beliefs in similar ways?

My hypothesis is that cultural contexts that bring about increasing emphasis on subjective well-being and gender equality could lead men to express comparatively less gender-biased expectations of altruism than women. That is, male in-group bias in the election of the dictator's gender is weaker for those who do not agree with the existence of different gender roles in society. Women's gender-biased expectations of altruism may be harder to change by socialization into different, more rational-secular and self-expressive moral values, because women's gender beliefs are less conditional upon those factors. Previous research on priming gender roles and gender stereotypes supports this hypothesis. Rudman and Phelan (2010) found that both traditional (e.g., a woman nurse) and non-traditional (e.g., a woman surgeon) primes resulted in low enthusiasm for masculine occupations. The authors explain this surprising effect because of the complex interaction between gender-role beliefs and gender-trait stereotypes. Whereas the traditional primes increased women's implicit communal stereotypes, which reduced their interest in masculine occupations; non-traditional primes decreased women's implicit leadership self-concept (i.e., they felt less competent and empowered). It has also been shown that adolescents' gender-role attitudes relate to gender-trait stereotypes in complex ways (Gibbons et al., 1991). Therefore, I speculate that male adolescents who are less traditional regarding gender roles also stereotype *less* with regard to male and female pro-social traits, i.e., male adolescents who disagree with the existence of different gender roles in society would not depict gender-biased expectations of altruistic behavior –i.e., they would describe less in-group bias in the election of the dictator's gender. Female adolescents, instead, would hold strong gender-trait stereotypes independent of their gender-roles beliefs.

Why judging gender roles as “natural” moderates male adolescents' gender beliefs but not those of female adolescents merits further investigation. What precise mechanism explains this gender difference in the current study remains obscure because the analyzed data is observational in nature. Additional experimental and cross-cultural research is needed to test this cognitive-cultural hypothesis on gender beliefs and social preferences. The results reported here are a good starting point for this further research.

## Ethics statement

This study was carried out in accordance with the recommendations of the National Commission of Scientific and Technological Research (CONICYT) of Chile, with written informed consent from all subjects parents and informed assent from all subjects. All subjects gave written informed consent in accordance with the Declaration of Helsinki. The protocol was approved by the Research Ethics Committee at the Universidad Andrés Bello.

## Author contributions

MS designed the study, analyzed the data, and wrote the final manuscript.

### Conflict of interest statement

The author declares that the research was conducted in the absence of any commercial or financial relationships that could be construed as a potential conflict of interest.

## Footnotes

^1^Instructions (translation to English) are available on the journal web page as Supplemental Material. Instructions and activities were like Aguiar, et al.'s study (2009).

^2^Although gift-cards to popular shops are acceptable substitutes to monetary incentives for adolescents, some schools where I performed the experimental sessions were in rural areas, far away from urban centres where popular shops are present. Due to health concerns, I tried to choose (when possible) a mix of sugar-free and low-fat snacks.

^3^It is important to notice that by estimating the likelihood of adolescents of choosing the women box, I am estimating whether adolescents manifested gender-biased expectations of altruism in choosing the boxes: if adolescents are biased to choose the box that is like their own sex, female adolescents should be more likely to choose the women box than male adolescents.

^4^Priming means that certain associations are activated in the mind just before a task is to be undertaken. For an investigation of this gender priming effects in DG, see (Boschini et al., 2012).

## Appendix

**Table A1:** Socio-demographic variables.

**Variable**	**Question**	**Categories/range**	**Mean/proportion**	***SD***
Gender		0) Male; 1) Female	0.42 (female)	–
Ethnicity	Do you belong to an ethnic group? (Yes/No) / If yes, “Which one?”	1) Mapuche; 2) Aymara; 3) Rapa Nui; 4) Likan Antai; 5) Quechua; 6) Colla; 7) Diaguita; 8) Kawéskar; 9) Yagán o Yámana; 10) Another one; 11) Do not belong to any ethnic group [recoded into a dummy variable for the analyses: 0) No, 1) Yes]	0.14 (Amerindian)	–
Risk-seeking score		0–6	3.51	1.77
Age		15–20	16.93	1.17
Single-gender school	Is this a single-gender school? [Information obtained from the Chilean Ministry of Education]	0) No; 1) Yes	0.58	–
School SES	Participants' socioeconomic status	0) Lowest; 1) Medium-Low; 2) Medium; 3) Medium-High; 4) Highest	0.06 0.14 0.35 0.29 0.17	–
Gender Roles	Men and women each have different roles to play in society	0) Strongly disagree; 5) Strongly agree	1.89	1.78

*Variable descriptions (translated to English from Castilian Spanish)*.
