# Supplementary material for: Gender-Biased Expectations of Altruism in Adolescents
Source: Front Psychol. 2018 Apr 10;9:484. doi: 10.3389/fpsyg.2018.00484 (PMC5902534; doi:10.3389/fpsyg.2018.00484)
Supplement: Supplementary file 1 [file Image1.PDF]

# EXPERIMENTAL INSTRUCTIONS:
# ADOLESCENTS' GENDER BELIEFS OF ALTRUISM IN DICTATOR GAMES

## INTRODUCTION

Welcome!

The purpose of this study is to explore how individuals make decisions in certain contexts. The instructions are very simple, and if you follow them carefully you will earn a number of coins. This will be done in a confidential way, so no one will know how many coins the other participants have received. At the end of the study, the three participants with the most coins will win a snack bag. If you have any questions, please raise your hand. Aside from these questions, you are not allowed to communicate with the other participants in the study.

The study consists of two phases that will be described in greater detail in a moment. In each phase, you will play a simple game.

## FIRST STAGE: FOUR.-ROUND DICTATOR GAME

In this stage, you will play a simple game of four rounds. To play this game, in each round you will be randomly paired with another participant in the room. Therefore, each pair consists of two players, A and B. In each round, you will play either the role of player A or B. The role you play in each round will be also randomly assigned by the computer system. Player B will receive 10 coins, and she/he will have to assign the 10 coins between herself/himself and Player A. Player B is allowed to assign to Player A any amount of coins, from 0 coins to 10 coins. Player A just has to wait until the division of the 10 coins by Player B is made. At the end of the round, as a reward, Player A will receive the amount of coins sent by Player B, and Player A will receive the amount she/he kept for herself/himself.

Before you begin the game, please respond these three simple questions to know your understanding of the game

1) How many coins will receive Player B at the beginning of the game?
2) What is the minimum amount of coins that Player B can assign to Player A?
3) Can Player A reject the amount of coins given by Player B?

## SECOND STAGE: GUESSING TASK

Now you will see two boxes in the screen. One of the boxes is labelled "women" and the other is labelled "men". Each box contains twenty assignations made by school pupils like you who were Players B in the same game you just played, but from a different school. Thus, each of the twenty assignations correspond to the number of coins, from 0 to 10, that these pupils in question gave to the other player.

Your decision involves choosing one of the two boxes earn one of the Player B's assignation. You simply choose the box you prefer, and the computer system will randomly pick out one assignation

from that box. You will earn the number of coins that corresponds to the assignation the computer system picks out.
